# Adaption to High Altitude: An Evaluation of the Storage Quality of Suspended Red Blood Cells Prepared from the Whole Blood of Tibetan Plateau Migrants

**DOI:** 10.1371/journal.pone.0144201

**Published:** 2015-12-04

**Authors:** Rui Zhong, Hua Liu, Hong Wang, Xiaojuan Li, Zeng He, Meiduo Gangla, Jingdan Zhang, Dingding Han, Jiaxin Liu

**Affiliations:** 1 Institute of Blood Transfusion, Chinese Academy of Medical Sciences and Peking Union Medical College, Chengdu, Sichuan, China; 2 Tibet Autonomous Region blood center, Lhasa, Tibet, China; German Red Cross Blood Service Frankfurt, GERMANY

## Abstract

Hypoxia has been reported to cause the significant enhancement of hemoglobin (Hb) and hematocrit (Hct), which stabilizes at relatively high levels after an individual ascends to a high altitude. However, the quality of the suspended red blood cells (SRBCs) obtained from individuals at high altitudes such as Tibetan plateau migrants after storage has not been studied. In this study, we compared the storage quality of SRBCs prepared from Tibetan plateau and Deyang lowland populations by adding a normal volume of mannitol-adenine-phosphate (MAP), which is a common additive solution used in blood storage in Asian countries. The storage cell characteristics were examined on days1, 7, 14 and 35.We found higher Hct and Hb levels and viscosity in the high altitude samples. The metabolic rates, including those for electrolytes and lactate, were higher in plateau SRBCs than in lowland SRBCs; these findings were consistent with the higher osmotic fragility and hemolysis of plateau SRBCs throughout the entire storage period. In addition, the reduction rates of 2,3-diphosphoglycerate (2,3-DPG) and oxygen tension to attain 50% oxygen saturation of Hb (P50) in plateau SRBCs were higher than those in lowland SRBCs, and the oxygen delivering capacity in plateau SRBCs was weaker than that in lowland SRBCs. We concluded that the storage quality of plateau SRBCs was inferior to that of lowland SRBCs when using the same concentration of MAP. We suggested that the optimal formula, including the MAP concentration or even a new additive solution, to store the plateau SRBCs must be assessed and regulated.

## Introduction

Environmental conditions such as high altitude affect blood cell morphology and phenotype [1–3]. Hypoxia in high altitude areas causes red blood cell (RBC), hemoglobin (Hb) and hematocrit (Hct) levels to significantly increase after individual persons ascend to high altitudes, and the enhancements stabilize at relatively high levels [4]. Han Chinese who migrated to the Qinghai-Tibetan Plateau had higher Hb concentrations than native Tibetans due to decreased plasma volume in the early arrival days and to increased numbers of red blood cells in the later days after arrival [5,6]. The increased Hb is potentially important for improving the oxygen-carrying capacity of RBCs. However, Hct values over 50% to 55% increase the risk in clinical transfusion through increased blood viscosity [7].

Donated whole blood (WB) is usually separated into various products for various clinical transfusion purposes. Suspended red blood cells (SRBCs) are the most commonly transfused blood product [8,9]. Our previous study [10] showed that blood collected from residents migrating to Tibet was separated into SRBCs with the normal volume of the additive solution (AS) and that the Hct level was over 65%. However, the biological change and the clinical transfusion suitability of storing Tibetan plateau migrants’ SRBCs for up to 35 days have not been assessed. It has been shown that the SRBCs storage quality depends on the AS [11–13]. Mannitol-adenine-phosphate (MAP) is one of the most common AS in China and most Asian countries, and it enables the storage of SRBCs for up to 35 days following collection. Therefore, the aim of this study was to compare the quality between Tibetan plateau migrants’ SRBCs and Deyang lowland residents’ SRBCs stored using a normal volume of MAP.

In this study, we collected WB from the Tibet Autonomous Region blood center (elevation 3650 m) and Deyang Blood Center (elevation 400 m). SRBCs were prepared by removing plasma and adding MAP. The separated SRBCs units were stored under standard blood banking conditions at 4°C. The samples were examined aseptically on Days 1, 7, 14, and 35 post-storage. The following parameters were tested: extracellular pH, electrolytes, crystal osmotic pressure, glucose, lactate, adenosine triphosphate (ATP), 2, 3-diphosphoglycerate (2,3-DPG), hemolysis rate, osmotic fragility, and oxygen affinity.

## Materials and Methods

### Whole blood collection, RBC processing, and storage

WB was collected according to standard procedures. We harvested the WB of 8 volunteer donors at the Tibet Autonomous Region blood center and of 8 lowland volunteer donors at Deyang Blood Center. All donors from Tibet were Han Chinese who migrated to the Tibetan plateau for more than one year. The 16 donors were male, and their ages ranged from 19–40. The information of weight and height of the donors was not supplied by the blood centers. They were regular donors, and this is the first time for them to donate. The study protocol was approved by the Institute of Blood Transfusion Ethics Committee. WB (400 mL ±10%) was collected into blood collection packs containing citrate-phosphate-dextrose-adenine-1 (CPDA-1) anticoagulant (56mL) (Nan Geer Biomedical Corporation, Sichuan, China). The formulation of the anticoagulant CPDA-1 was listed in Table 1. Each bag of WB was centrifuged at 3500 ×g for 15 minutes at 4°C. The plasma was removed, and 100 mL MAP (see Table 1 for the additive solution composition) was added to the packed RBCs to obtain the SRBCs. The SRBCs units were stored under standard blood banking conditions at 4°C and were analyzed on days1, 7, 14, and 35 of storage.

**Table 1:** CPDA-1 and MAP Composition.

Composition	CPDA-1	MAP
Sodium citrate (mM)	89	5.1
Acidcitrate (mM)	17	1
Sodiumphosphate (mM)	5.8	_
NaH_2_PO_4_ (mM)	_	7.8
Glucose (mM)	177	40
NaCl (mM)	_	85
Adenine (mM)	2	1
Mannitol (mM)	_	80

### Routine RBC quality assessment

On each day of sample collection, blood examinations were performed on an automated hematology analyzer (BC-5800; Mindray, Shenzhen,China), and the RBC, Hb and Hct levels and mean corpuscular volume (MCV) were examined. Supernatant potassium (K^+^) and sodium (Na^+^) levels were measured on an electrolyte analyzer (HC-9885; Hangchuang Medical Instrument Co., Ltd, Shenzhen, China). Extracellular pH was measured with a pH meter (FE20; Mettler Toledo, Switzerland), crystal osmotic pressure was measured with an osmotic pressure instrument (SMC 30 C; Tianhe Medical Instrument Co., Ltd, Tianjin, China), and viscosity was assessed using a rheology instrument (990 BBT, South CNC Equipment Co., Ltd, Chongqing, China). RBC adenosine triphosphate (ATP) and 2,3-diphosphoglycerate (2,3-DPG) were assessed using commercial ATP and 2,3-DPG kits according to the recommended protocols (Roche Diagnostics, Mannheim, Germany). Plasma glucose was measured with a commercial glucose kit (Rongsheng Biotech Co., Ltd, Shanghai, China). The lactate concentration was analyzed in RBC protein precipitant extracts using lactate kits (Jiancheng Bioengineering Institute, Nanjing, China) adapted for use with a spectrophotometer.

### Scanning electron microscopy (SEM)

SEM studies of RBCs were performed using a Quanta 250electron microscope (FEI Company, Hillsboro, OR, United States). Blood samples were fixed in phosphate-buffered (pH 7.4) 2.5% glutaraldehyde for 2 h, mounted on mica slides, washed twice in 0.1 M phosphate buffer (pH 7.4), and then dehydrated in graded ethanol (50-70-80-90-100%). After drying with a lyophilizer (FD-1D-50, BrainLAB Kang Experimental Instrument Co., Beijing, China) in a vacuum chamber and covering with a gold-palladium layer, the samples underwent SEM analysis.

### Hemolysis

Briefly, free Hb was assessed by measuring the absorbance of cell supernatants at 435 nm using a spectrophotometer (Ultrospec 6300 Pro; Amersham Biosciences, United States) and correcting for the absorbance of plasma, if necessary. Hemolysis rate was expressed as a percentage of the total Hb present in RBCs after correcting for the Hct.

### Osmotic fragility

Osmotic fragility was assessed using a series of diluted sodium chloride solutions with concentrations ranging from 3.0 g/L to 9.0 g/L. Briefly, 30 μL of each RBC sample with 50% Hct was added to 3000 μL of each diluted sodium chloride solution and incubated at room temperature for 30 minutes, followed by centrifugation at 1000×g for 5 minutes. Supernatants were collected, and hemolysis was measured by a spectrophotometer at a wavelength of 540 nm. The percent hemolysis in each sodium chloride solution was plotted against the sodium chloride solution concentration to calculate the concentration that produced 50% hemolysis.

### Oxygen affinity

The oxygen tension to attain 50% oxygen saturation of Hb (P50) was detected by a Hemox analyzer (TCS Scientific, USA). To obtain a suspension, 50 μL RBCs were added to the diluents. Oxygen was to the suspension until the oxygen partial pressure (PO_2_) was greater than13.33 kPa (100 mmHg); oxygen was then stopped, and nitrogen was added until the PO_2_ was less than 0.67 kPa (5 mmHg). The changes in PO_2_ and oxy-hemoglobin were detected in the process, and oxygen dissociation curves (ODC) were drawn. Finally, P50 was measured according to the ODC.

### Statistical analysis

The results are shown as the mean ±standard deviation (SD) of eight sample replicates, and every parameter has to be test for at least 3 times, unless otherwise stated. Independent sample t tests were used to compare the RBCs from various areas stored for the same lengths of time and the P values were corrected by Bonferroni correction. Repeated measures analysis of variance (ANOVA) was used to assess the influence of storage time on lowland or plateau SRBCs. Data were analyzed using computer software (SPSS Statistics17.0, IBM SPSS, United States). The levels of significance were indicated in the text.

## Results

### Plateau SRBCs have higher RBC, Hb, and Hct levels and MCV than lowland SRBCs

At time of donation, we can’t get all the baseline values of the parameters except the concentration of hemoglobin. After the donation, the blood has to do virus detection, and we can’t have enough time and enough volume of blood to test all the parameters at time of donation. The hemoglobin of plateau and lowland blood was 165.62±10.15 and 137.43±7.38 g/L respectively. In China, leukodepletion was not asked for every blood center or hospital when preparing SRBCs, therefore, in this study, we didn’t use leukodepletion both for plateau and lowland blood. We have tested the number of leukocyte in plateau and lowland blood at beginning of storage (3.89±1.23 vs 4.52±0.89 ×10^9^/L), and there was no significant difference between the two groups.

**Fig 1:**
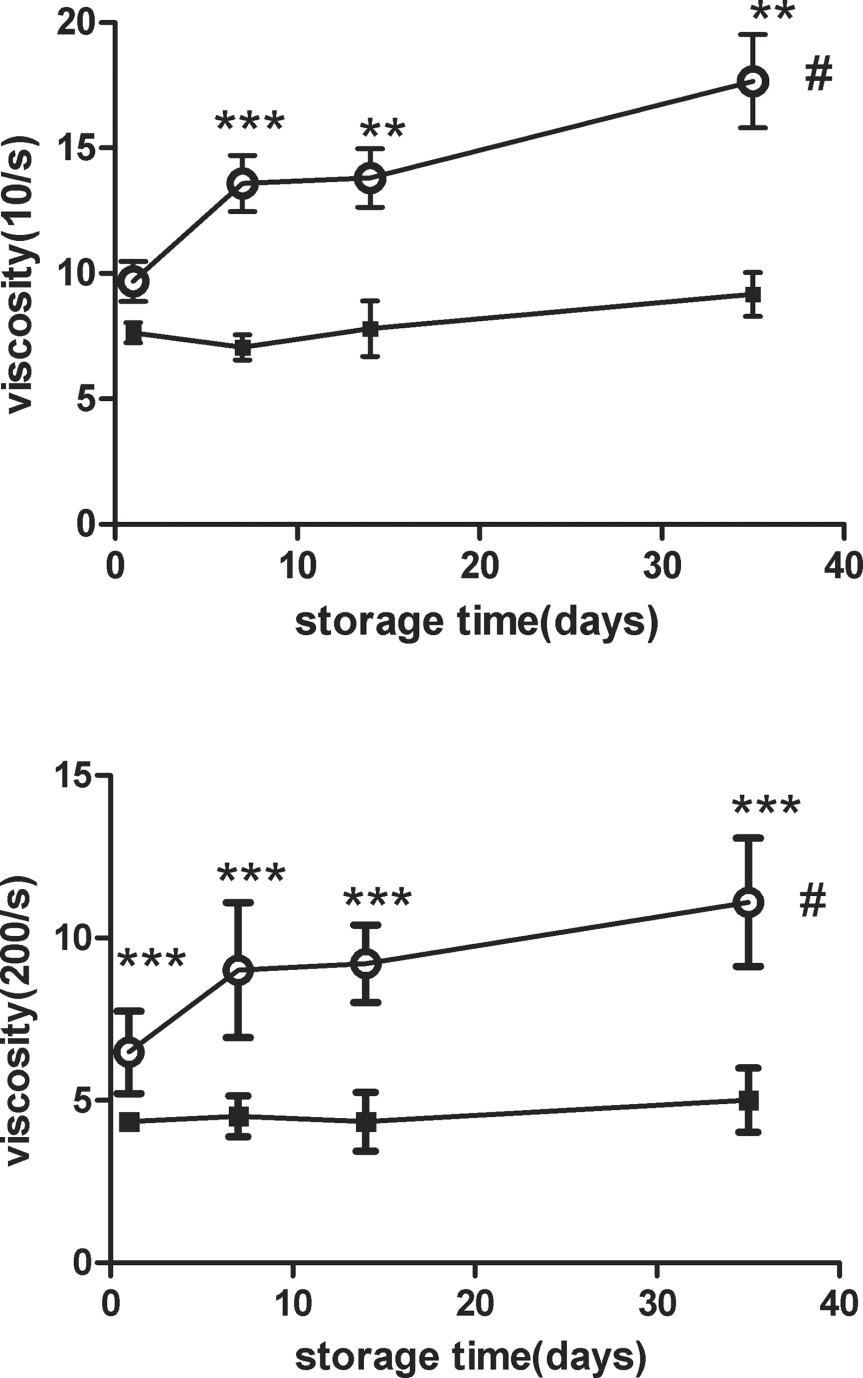
The viscosities of stored suspended red blood cells (SRBCs) from plateau (circle) and lowland (square) populations were measured throughout the 35-day storage period. The viscosity at ahigh shear rate (A) (***t test p < 0.001 for plateau vs. lowland from day1 to day 35). The viscosity at low shear rate (B) (***t test p < 0.001 for plateau vs. lowland on day7, **t test p < 0.01 for plateau vs. lowland from day14 today 35).# ANOVA p < 0.05 for the viscosities of plateau SRBCs at high and low shear rates throughout the entire storage duration.

On day 1 of storage, we found that Tibetan plateau SRBCs had significantly higher RBC, Hb, and Hct levels compared with Deyang lowland SRBCs (Table 2). We observed Hct enhancement in plateau SRBCs from 61.0% (day 1) to 64.3% (day 35), whereas the Hct value in lowland SRBCs had no significant relationship with the storage time. The MCV of plateau SRBCs also increased with the storage time, suggesting that the RBCs became more spherical (Table 3). The viscosities of plateau SRBCs at low and high shear rates both increased with storage time, and these values were higher than those of lowland SRBCs from day 7 to day 35 (Fig 1).

**Table 2:** The results of RBC numbers and Hb concentrations in the lowland (n = 8) and plateau groups (n = 8) on storage day 1.

RBC(×10^12^/L)		Hb(g/L)	
lowland	plateau	lowland	Plateau
6.02±0.33	6.99±0.66	173.51±9.62	202.03±16.72*

Data are reported as the mean±SD.

* t test p<0.05 for RBC and Hb levels in the plateau groupvs. the lowland group on day 1.

**Table 3:** Hct and MCV in the lowland (n = 8) and plateau (n = 8) groups throughout the entire storage period.

Storage Time (days)	Hct(%)		MCV(FL)	
	lowland	Plateau	lowland	plateau
1	45.7±5.2	61.0±2.4**	96.7±2.1	95.7±3.6
7	45.2±6.2	62.3±3.0**	97.3±2.0	96.4±3.0
21	44.2±3.8	63.3±3.4**	98.0±2.6	97.7±3.7
35	45.8±4.5	64.3±3.0** #	95.0±3.3	98.9±3.6* #

Data are reported as the mean±SD.

** t test p < 0.01 for Hct in the plateau group vs.the lowland group from day1 to day 35.

* t test p < 0.05 for MCV in the plateau group vs. the lowland group on day 35.

# ANOVA p < 0.05 for Hct and MCV in plateau SRBCs throughout the entire storage period.

### Plateau SRBCs have higher crystal osmolality, higher rates of change of electrolytes and lactate, and lower pH compared with lowland SRBCs

**Fig 2:**
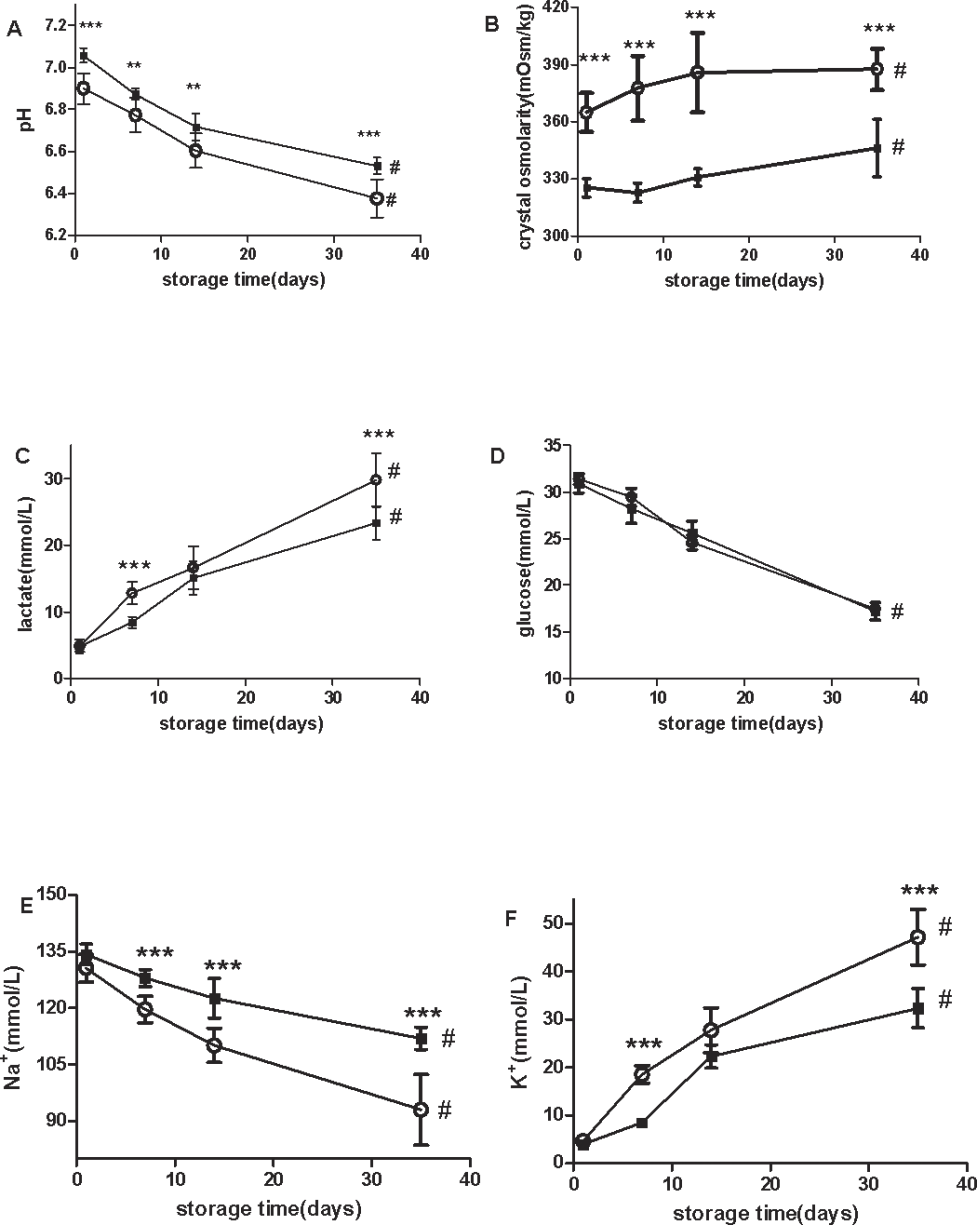
Standard quality parameters of stored suspended red blood cells (SRBCs) from plateau (circle) and lowland (square) populations were measured throughout the 35-day storage period. pH (A) (***t test p < 0.001 for plateau vs. lowland on days1 and 35), **t test p < 0.01 for pl eau vs. lowland on days 7 and 14).Crystal osmolarity (B) (***t test p < 0.001 for plateau vs. lowland from day1 to day 35).Lactate concentration (C) (***t test p < 0.001 for plateau vs. lowland on Days 7 and Day 35).Supernatant glucose (D) and Na^+^ (E)concentrations(***t test p < 0.001 for plateau vs. lowland on days7, 14 and 35).Supernatant K^+^ levels(F) (***t test p < 0.001 for plateau vs. lowland on days 7 and 35).# ANOVA p < 0.05 for all of the parameters throughout the entire storage duration.

The pH of the plateau SRBCs and lowland SRBCs was 6.9 and 7.05, respectively, on day1, and decreased to 6.4 and 6.6, respectively, by day35. Plateau SRBCs had a significantly lower pH compared with that of lowland SRBCs from day 1 to day 35 of storage (Fig 2A). In contrast, crystal osmotic pressure increased with storage time in both plateau and lowland SRBCs (Fig 2B). The glucose levels decreased with storage time in both plateau and lowland SRBCs, and no significant difference was found between the two groups (Fig 2D). The production of lactate was higher in plateau SRBCs than lowland SRBCs on days 7 and 35 of storage (p< 0.05; Fig 2C). Plateau SRBCs had higher K^+^ levels in the supernatant compared with lowland SRBCs on days 7 and day 35, whereas lowland SRBCs had higher Na^+^ levels in the supernatant compared with plateau SRBCs (Fig 2E & 2F).

We found that the RBC count in plateau SRBCs was much higher than that in lowland SRBCs and that the RBC metabolism parameters such as glucose, lactate, Na^+^ and K^+^ were correlated to the RBC number. To exclude the influence of RBC number on these parameters, we therefore propose the following formula to calculate the rate of RBC metabolism:

$$R_{x}=\frac{V_{x1}-V_{x35}}{N_{rbc}}$$

where R represents the change rate of these parameters throughout the storage period, x represents the parameters, including glucose, lactate, Na^+^ and K^+^,V_x1_ represents the value of the parameters on day 1 of storage,V_x35_ represents the value of parameters on day 35 of storage, and N_rbc_ represents the number of RBCs on day 1 of storage.

The results (Table 4) revealed a significant difference between plateau and lowland RBCs during the entire storage period for the change rates of these parameters, with the exception of glucose, although the plateau glucose level was still higher than the lowland glucose level.

**Table 4:** The change rates of Na^+^, K^+^, glucose and lactate in SRBCs throughout the storage period.

SRBCs units	Na^+^	K^+^	Glucose	Lactate
	mmol/L/rbc×10^12^	mmol/L/rbc×10^12^	mmol/L/rbc×10^12^	mmol/L/rbc×10^12^
Lowland	3.68±0.15	4.72±0.21	1.68±0.16	3.07±0.51
plateau	5.38±0.25**	6.07±0.30**	1.78±0.21	3.56±0.53*

Data are reported as the mean±SD.

* Significant results (p<0.05) for lactate in plateau SRBCs compared with lowland SRBCs.

** Significant results (p<0.01) for Na^+^ and K^+^ in plateau SRBCs compared with lowland SRBCs.

### RBC morphology images

**Fig 3:**
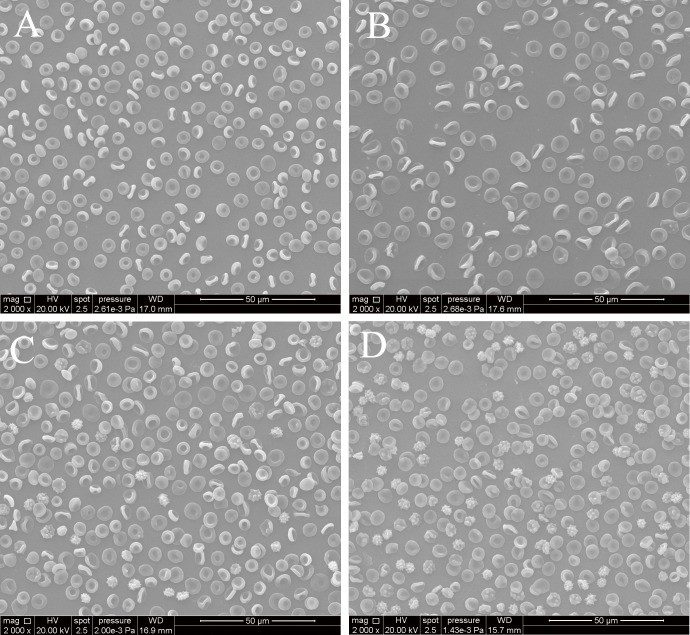
Scanning electron microscopy of stored red blood cells (RBCs). RBCs isolated from stored lowland RBCs on day1(A) andday35(C) and from stored plateau RBCs on day1(B) and day 35(D); magnification:2000×.

SEM studies showed a significant RBC shape transformation during blood storage. On day 1, discocytes were the dominant cell population in both lowland and plateau SRBCs (Fig 3A). On day 35, the number of normally shaped discocytes among plateau SRBCs was much less than among lowland SRBCs, and the number of abnormally shaped RBCs of the plateau group continued to increase with storage time (Fig 3B).

### Plateau SRBCs have higher 2,3-DPG and P50 at the beginning of storage, and no significant difference exists for ATP between plateau and lowland SRBCs

**Fig 4:**
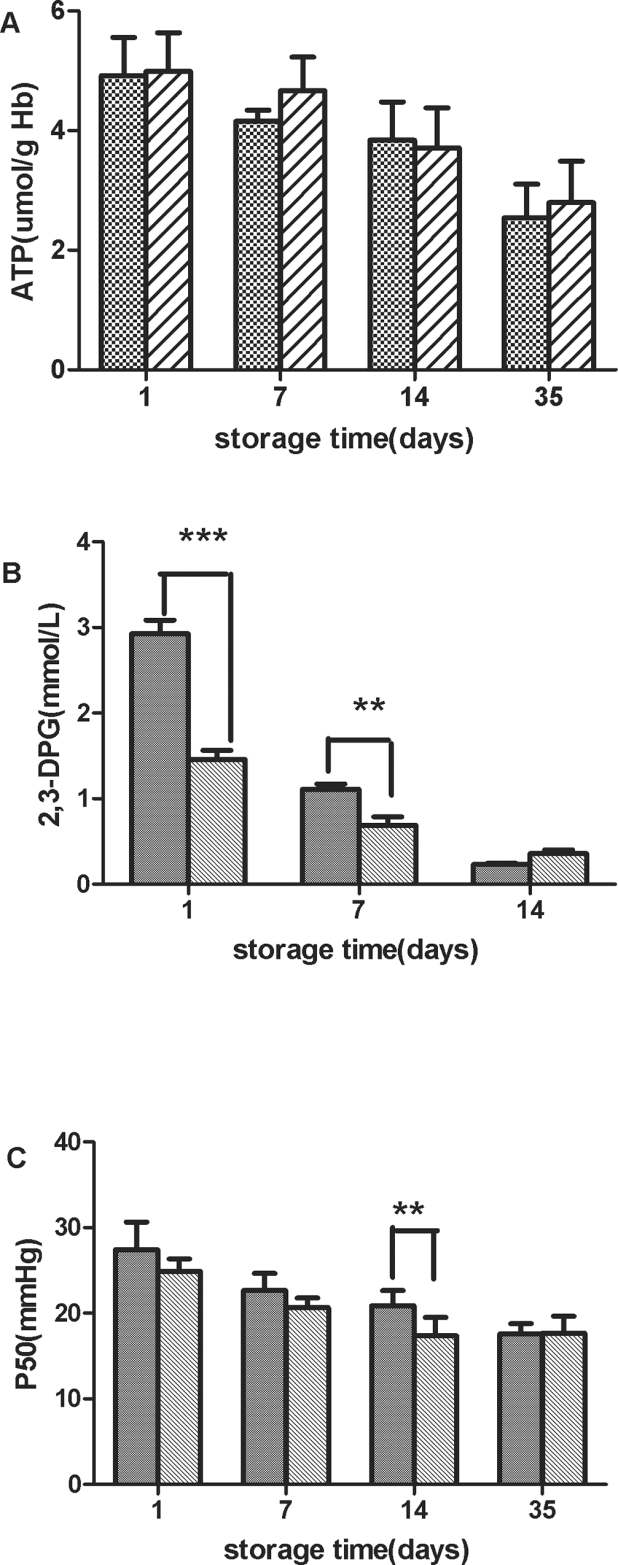
The adenosine triphosphate (ATP) and2,3-diphosphoglycerate (2,3-DPG) levels and oxygen tension to attain 50% oxygen saturation of Hb (P50) of stored RBCs from plateau (square) and lowland (stripe) populations were measured throughout the 35-day storage period. ATP (A) 2,3-DPG (B)(***t test p < 0.001 for plateau vs. lowland on day 1, **t test p < 0.01 for plateau vs. lowland on day 7). P50 (C)(**t test p < 0.01 for plateau vs. lowland on day 14).

No significant difference in ATP levels was found between the two groups (Fig 4A). 2,3-DPG levels in plateau SRBCs were higher than those in lowland SRBCs from day 1 to day7 (p < 0.01), whereas no significant difference was found on day 14 (Fig 4B).

When the SRBCs of the two groups were stored, there was a prompt rise in oxygen affinity, as shown in Fig 4C by a decrease in P50, the partial pressure of oxygen at which Hb is half saturated. Plateau SRBCs had significantly higher P50 values than lowland SRBCs on day 14, but no significant difference was found between the two groups at the end of the storage period.

### Plateau SRBCs have higher osmotic fragility and hemolysis than lowland SRBCs

**Fig 5:**
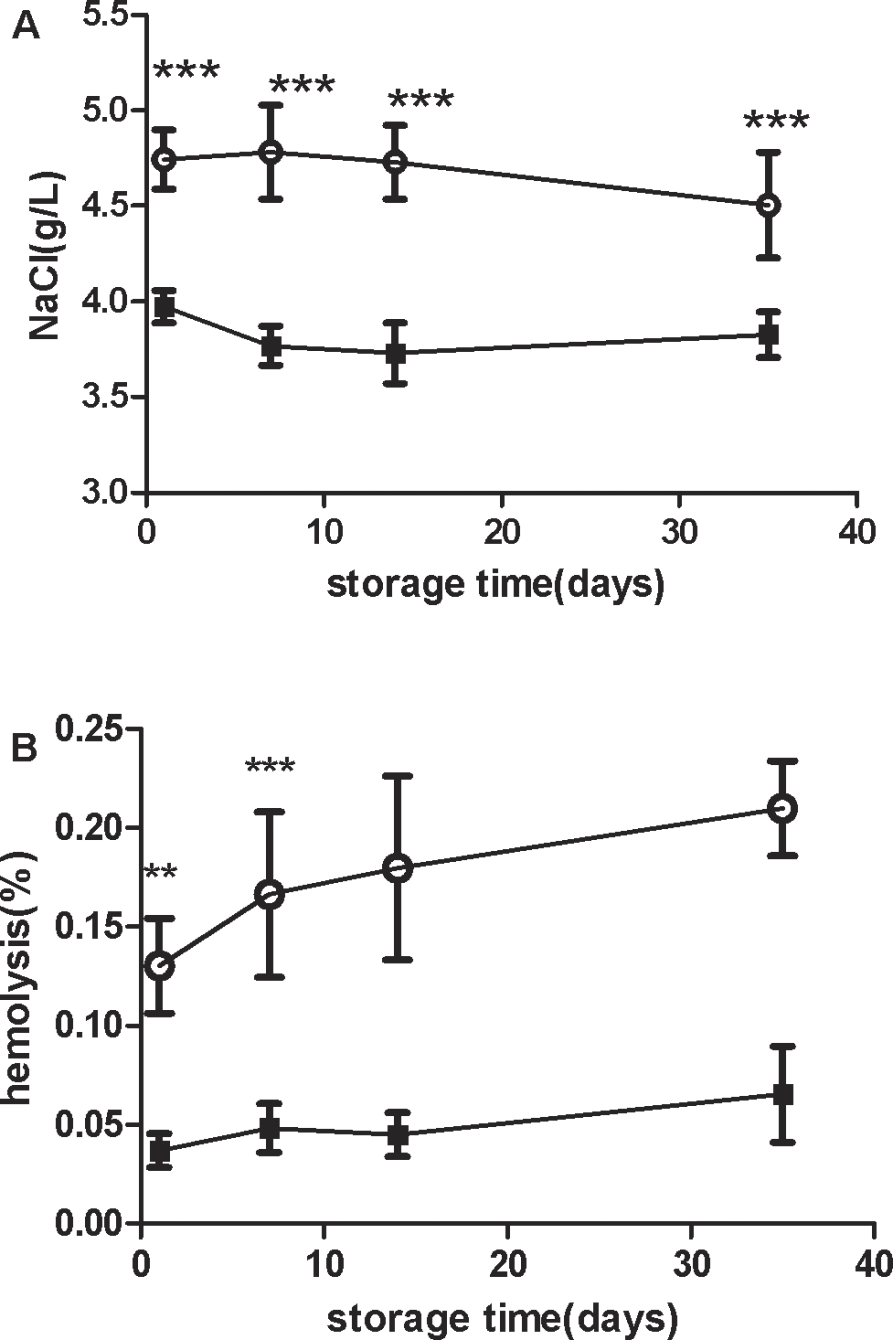
The osmotic fragility and hemolysis of stored suspended red blood cells (SRBCs) from plateau (circle) and lowland (square) populations were measured throughout the 35-day storage period. Osmotic fragility (A) (***t test p < 0.001forplateau vs. lowland from day 1 to day35). Hemolysis (B) (**t test p < 0.01 for plateau vs. lowland on day1, ***t test p < 0.001 for plateau vs. lowland on day7).

The plateau SRBCs were less resistant to osmotic stress than the lowland SRBCs. Whereas 50% of the plateau RBCs lysed at a NaCl concentration of 4.75 ± 0.25 g/L, the lowland RBCs were more resilient, lysing at a concentration of 3.81 ±0.15 g/L(Fig 5A). Although the hemolysis was within the COE acceptance limit of 0.8% throughout the storage of all RBC units (Fig 5B), the value for plateau SRBCs was higher than that for lowland SRBCs on day 1 and day 7, which was consistent with the results for osmotic fragility.

## Discussion

In this study, we compared the quality of SRBCs from migrants to the Tibetan Plateau with those from lowland residents during a 35-day storage period. Our findings suggested that the storage quality of plateau SRBCs was worse than that of lowland SRBCs when using the same volume of MAP. Besides, the amount of citrate in the primary donation bag was identical in the two groups, and we didn’t see any differences in clot formation during storage just from the observation by eyes, and the difference in blood coagulation between the two groups needs further study.

At high altitudes, in response to the insufficient amount of oxygen, the bone marrow is stimulated by an erythropoietic factor to increase RBC production [14,15]. In addition to the increase in RBCs, the Hb in plateau SRBCs is augmented. Our data showed that the average Hb in plateau SRBCs ranged from 185 to 218 g/L, which was increased compared with the lowland SRBCs levels, ranging from 164 to 183 g/L at sea level, similarly to the findings of previous studies [16–18]. Some investigators believe that increased RBC number is a necessary adaptation characteristic, whereas others suggest that better-adapted natives have lower Hct [19]. It was suggested that our findings of higher RBC and Hb levels in SRBCs from plateau migrants living at a high altitude for more than one year were indications of new adaptation features in these people.

Similarly, the MCV in plateau SRBCs also increased with storage time, and the MCV was higher in plateau SRBCs than in lowland SRBCs at the end of storage, indicating that plateau RBCs became swollen and spherical during storage. The gradual increase in MCV in plateau SRBCs over time might result from insufficient ATP-dependent Na-K pump activity at the RBC membrane, as these pumps cannot work properly under cold storage conditions [20]. The SEM findings of plateau RBCs were consistent with the MCV on day 35 of storage, and the number of abnormally shaped plateau RBCs was much higher than that of lowland RBCs. According to Bessis’ and Brezina’s classification and discrimination between reversible and irreversible membrane shape alterations over the storage duration [21,22], the number of reversible shapes such as echinocyte and stomatocyte shapes in plateau RBCs was much lower than that of lowland RBCs on day 35 of storage, and the percentage per500 cells was 68% vs. 75%, respectively. In contrast, the number of irreversible shapes such as spheroechinocyte and spherostomatocyte shapes was higher in plateau RBCs compared with lowland RBCs. Previous studies indicated that RBC shape changes are related to enhanced osmotic fragility and cation dysregulation, and are accompanied with increasing hemolysis and acidosis [23,24]. Our findings in this study were consistent with a previous study indicating that higher osmotic fragility, hemolysis, plasma K^+^ accumulation and lower pH of plateau SRBCs may cause the abnormal shape changes in plateau RBCs in our storage conditions.

Additionally, RBC shape was also influenced by other factors, including deformability and rheological properties [25]. The higher viscosities of plateau SRBCs at high and low shear rates may be caused by the higher Hct and Hb of plateau SRBCs and are also associated with the higher MCV and decreased RBC deformability [26]. Additionally, the viscosity of stored plateau SRBCs increased with storage time, indicating that the higher viscosity of plateau SRBC units at the end of their shelf-life increases the difficulty of clinical transfusion. Therefore, blood with an Hb concentration higher than 180 g/L was not collected by the Tibetan blood center.

A previous study found that that the initial pH value affected RBCs throughout the entire storage period [27]. Our study showed that from day 1 to day 35, the pH of plateau SRBCs was significantly lower than that of lowland SRBCs and was associated with continuous glucose metabolism, subsequently causing an accumulation of lactic acid and a stronger inhibition of glycolysis. Tighter packing of RBCs led to a faster rate of glucose depletion and more difficulty in in vivo recovery [28,29]. These findings suggested that the lower pH of plateau SRBCs was associated with higher Hct.

Plateau SRBCs had higher extracellular K^+^ levels compared with those of lowland SRBCs during storage, whereas the Na^+^ levels in plateau SRBCs supernatant were lower. Higher extracellular K^+^ indicates that the intracellular K^+^ loss rate was faster in plateau RBCs than in lowland RBCs. The loss of K^+^ from RBCs causes dehydration, resulting in shape changes and rheological defects [30].The crystal osmotic pressure of plateau SRBCs was higher than that of lowland SRBCs and increased gradually during storage; the increased crystal osmotic pressure maybe associated with the increased K^+^ leakage of plateau SRBCs. The results of the higher change rates of Na^+^, K^+^ and lactate production in plateau SRBCs suggested that these parameters were caused not only by Hct and Hb but also by RBC properties, including osmotic fragility and hemolysis.

Osmotic fragility analysis indicated that lowland SRBCs had greater osmotic resilience compared with plateau SRBCs. A previous study suggested that the enhancement of osmotic fragility increased cell sphericity and cell volume, which was corroborated by a higher MCV in plateau SRBCs [31]. These findings were consistent with our hemolysis data, and the higher osmotic fragility of plateau SRBCs was in agreement with the higher spontaneous hemolysis rate after storage compared with that of lowland SRBCs. Therefore, the capacity to withstand osmotic stress was worse. We guess the deformability of plateau RBC would be worse and the RBC may not pass through capillaries smoothly when the plateau blood transfusion into recipients’ body, thus, the oxygen of recipients’ organ may not be enough. On the other hand, the half life of plateau RBC may be shorter than lowland RBC after it enters into the body. However, all the tests in the study are in vitro, and more transfusion outcomes need further study. Both values of hemolysis of lowland and plateau (0.066±0.068% and 0.18±0.13%, respectively) SRBCs on day 35 of storage are, however, well within the allowances of 0.8% in Europe and 1.0% in North America [32].

ATP concentrations in both lowland and plateau SRBCs gradually decreased throughout storage. As the ATP concentrations decrease, the Na/K membrane pumps begin to fail, and intracellular K^+^ is leaked into the storage media [33].However, no significant difference in the ATP concentration between lowland and plateau SRBCs was found throughout the entire storage period, which was not consistent with the results of higher K^+^ leakage in plateau SRBCs. Although the loss of ATP stiffens the RBC membrane due to calcium accumulation [11], we propose that the differences of K^+^, Na^+^ and lactate concentrations in lowland and plateau SRBCs were not primarily caused by energy metabolism but rather by inherent differences in RBC membrane fragility at the beginning of storage [34].

At high altitudes, the affinity of Hb for oxygen is decreased to make Hb-bound oxygen more available to body tissues [35].In 1960s, Lenfant and Torrance studied the relationship between oxygen dissociation and RBC organic phosphate concentrations in subjects who moved from one altitude to another and found that 2,3-DPG and P50 both increased and that 2,3-DPG formed a complex with purified deoxyhemoglobin. This combination reduced the affinity of Hb for oxygen when subjects moved from lowland to plateau [18].In comparing the results of 2,3-DPG and P50 in plateau SRBCs to those of lowland SRBCs, the data reported here showed similar trends as a previous report, indicating that the plateau SRBCs had higher 2,3-DPG and P50 values at the beginning of storage. However, no significant differences in 2,3-DPG and P50 levels were found between lowland and plateau RBCs at end of storage, suggesting that the reduction rates of 2,3-DPG and P50 in plateau SRBCs were greater than those of lowland SRBCs and that the oxygen-delivering capacity of stored plateau SRBCs was weaker compared with that of lowland SRBCs.

## Conclusions

In conclusion, we found an important difference in the storage quality of SRBCs from Tibetan Plateau migrants and Deyang lowland residents. Altitude may play a major role in these changes. In addition to the higher Hct and Hb levels and viscosity induced by the high altitude, the rates of electrolyte and lactate metabolism of plateau SRBCs were higher than those of lowland SRBCs during storage. These findings were consistent with the higher osmotic fragility and hemolysis of plateau SRBCs throughout the entire storage period. We concluded that the storage quality of plateau SRBCs was inferior to lowland SRBCs when using the same volume of the additive solution MAP. We recommend increasing the volume of AS for preparing plateau SRBCs, which can reduce the concentration of Hb and viscosity of blood for the convenience of clinical transfusion, or regulating the formula of the storage solution, for example, adding ascorbic acid to improve the membrane fragility of RBC, or finding a new suitable alternative solution for plateau SRBCs.
